# Self-Assembly and Release of Peste des Petits Ruminants Virus-Like Particles in an Insect Cell-Baculovirus System and Their Immunogenicity in Mice and Goats

**DOI:** 10.1371/journal.pone.0104791

**Published:** 2014-08-12

**Authors:** Wenchao Li, Hongyan Jin, Xiukun Sui, Zhanzhong Zhao, Chenghuai Yang, Wenquan Wang, Junping Li, Gang Li

**Affiliations:** 1 State Key Laboratory of Animal Nutrition, Beijing Institute of Animal Science and Veterinary Medicine, Chinese Academy of Agricultural Sciences; Beijing Scientific Observation and Experiment Station for Veterinary Drug and Veterinary Biotechnology, Ministry of Agriculture, Beijing, PR China; 2 China Institute of Veterinary Drug Control, Beijing, PR China; 3 Beijing CevaHuadu Biological Co., Ltd, Beijing, PR China; The University of Texas Medical Branch, United States of America

## Abstract

Peste des petits ruminants (PPR) is an acute, febrile, viral disease of small ruminants that has a significant economic impact. For many viral diseases, vaccination with virus-like particles (VLPs) has shown considerable promise as a prophylactic approach; however, the processes of assembly and release of peste des petits ruminants virus (PPRV) VLPs are not well characterized, and their immunogenicity in the host is unknown. In this study, VLPs of PPRV were generated in a baculovirus system through simultaneous expression of PPRV matrix (M) protein and hemaglutin in (H) or fusion (F) protein. The released VLPs showed morphology similar to that of the native virus particles. Subcutaneous injection of these VLPs (PPRV-H, PPRV-F) into mice and goats elicited PPRV-specific IgG production, increased the levels of virus neutralizing antibodies, and promoted lymphocyte proliferation. Without adjuvants, the immune response induced by the PPRV-H VLPs was comparable to that obtained using equivalent amounts of PPRV vaccine. Thus, our results demonstrated that VLPs containing PPRV M protein and H or F protein are potential “differentiating infected from vaccinated animals” (DIVA) vaccine candidates for the surveillance and eradication of PPR.

## Introduction

Peste des petits ruminants (PPR) is a highly contagious and economically important viral disease of domestic and some wild small ruminants, and in particular, of goats and sheep. It is notifiable to the Office International des Epizooties (OIE). Clinically, the disease is characterized by severe pyrexia, oculonasal discharges, necrotizing and erosive stomatitis, enteritis, and pneumonia. It was first described in the Ivory Coast, West Africa, but has now become wide-spread in sub-Saharan Africa, Arabia, the Middle East, Southwest Asia, India, and other countries[1]. In China, PPR was first reported in Tibetin 2007[2], and in December 2013, a PPR outbreak was reported in Xinjiang Yili; in this outbreak,1236 goats were infected, of which 203 died, and6671 goats in the susceptible population were killed [3]. Thus, PPR outbreaks can cause severe economic losses, because they often result in high morbidity and mortality; therefore, development of an effective vaccine for the prevention and control of PPR is particularly important.

The causative agent, PPR virus (PPRV), is a member of the family Paramyxoviridae and the genus *Morbillivirus* [4].The non-segmented, single stranded, negative-sense RNA genome of PPRV encodes six structural proteins (nucleocapsid [N], phosphoprotein [P], matrix [M], fusion [F], hemagglutinin [H], and polymerase [L] proteins), and two non-structural proteins(C and V). Proteins N, P, and L are required for reconstituting viral RNA polymerase activity; M protein is required for particle formation and budding, and the two surface glycoproteins, H and F, are required for attachment and entry into the host cell. Structurally, the virions are morphologically pleomorphic particles that are surrounded by a host-derived membrane, modified by the H and F transmembrane glycoproteins, as well as the M protein, which is associated with the inner surface of the viral membrane[5], [6].As in other paramyxoviruses, the C protein of PPRV is expressed from an alternative open reading frame (ORF) within the P gene, whereas the V protein is expressed by RNA editing [7]. These two proteins are thought to be involved in viral evasion of the host's immune responses, specifically by blocking interferon production during infection [8].

For the control of PPR, four attenuated vaccines are available commercially. In endemic areas, the virus is currently controlled using the Nigeria 75/1 strain (Nig75/1), which has been attenuated by serial passage in Vero cells. In India, three other live attenuated vaccines are currently licensed for use: Sungri 96, Arasur 87, and Coimbatore 97 [9]. However, a major disadvantage of these attenuated vaccines is that they cannot be used for differentiating infected from vaccinated animals(DIVA).Recently, subunit vaccines have been developed based on the incorporation of PPRV immunogens into various vector systems, such as sheep or goat pox, vaccinia virus, adenovirus, or baculovirus vectors [10], [11], [12], [13].

Virus-like particles (VLPs) recently emerged as alternatives to subunit vaccines; these have the advantage that they mimic the organization and conformation of the native virus capsid and envelope, but lack the viral genome [14], [15].Given their intrinsic immunogenic properties and high safety profile, VLPs have been generated in different heterologous expression systems and have been tested as vaccine candidates for a variety of viral diseases [16]. In the human vaccines market, six VLP-based vaccines, including three for hepatitis B (Recombivax HB, Engerix B, and GenHevac B), two for human papillomavirus (Gardasil and Cervarix), and one for influenza A (InflexalV), have been developed [16], [17].In the veterinary field, a VLP-based vaccine against porcine circovirus type 2 (PCV2), viz., Porcilis PCV, Intervet, has recently been licensed [18].

To date, the assembly and release of PPRV VLPs have not been well characterized, and their immunogenicity remains unknown. It is known that the PPRV glycoproteins H and F contain neutralizing antibody epitopes and several T cell epitopes [19], [20], and that PPRV-specific lympho proliferative responses can be elicited in goats immunized with a recombinant adenovirus expressing the H or F protein[10]. Therefore, we hypothesized that immunization with VLPs containing H or F proteins could induce a strong PPRV-specific immune response.

In this study, two types of VLPs for PPRV (PPRV-H and PPRV-F)were developed, using viral M protein co-expressed with H or F protein. Mice and goats immunized subcutaneously with these PPRV VLPs developed both humoral and cellular immune responses.

## Materials and Methods

### Cell culture and viruses

PPRV vaccine strain (Nig75/1) was obtained from the China Institute of Veterinary Drug Control, Beijing, China. Vero cells (The American Type Culture Collection, ATCC: CCL-81, Manassas, VA, USA) were cultured in Dulbecco's modified Eagle medium (DMEM; Invitrogen, Grand Island, NY, USA) containing 10% fetal bovine serum (FBS; Invitrogen, Grand Island, NY, USA) at 37°C with 5% CO_2_, and were used for PPRV propagation and titration. *Spodoptera frugiperda* Sf21 insect cells were maintained in Sf-900II insect serum-free medium (Invitrogen, Grand Island, NY, USA) as monolayer cultures or in suspension cultures maintained on temperate orbital shakers (120rpm) at 28°C.

### Cloning of M, H, and F genes and construction of bacmid transfer plasmids

PPRV RNA was extracted using Trizol LS (Invitrogen, Carlsbad, CA, USA). Reverse transcription(RT) and polymerase chain reaction (PCR) were performed on extracted viral RNA with gene-specific oligonucleotide primers(Table 1) that had been designed according to the sequence of the PPRV Nig75/1 strain (GenBank accession no. X74443.2).Following RT-PCR, the cDNA fragments containing the PPRV M, H, and F genes were cloned into the pMD18-T vector (Takara, Dalian, China). The integrity of the nucleotide sequences of the M, H, and F genes was verified by DNA sequencing.

**Table 1:** Primer sequences used for RT-PCR amplification of M, H, and F genes of peste des petits ruminants (PPRV) and identification of recombinant bacmids.

Primers	Primers Sequence (5′–3′)	Enzyme site
M1	CCGCTCGAGGCCACCATGACCGAGATCTAC	*Xho*I
M2	CGGGGTACCTTACAGGATCTTGAACAG	*Kpn*I
H1	CGGGATCCGCCACCATGTCCGCACAAAGG	*Bam*HI
H2	CCCAAGCTTTCAGACTGGATTACATGTT	*Hin*dIII
F1	CGCGGATCCGCCACCATGACACGGGTCG	*Bam*HI
F2	GCTCTAGACTACAGTGATCTCACGTAC	*Xba*I
M13 For	CCCAGTCACGACGTTGTAAAACG	
M13 Rev	AGCGGATAACAATTTCACACAGG	

The primers used for RT-PCR amplification of the M, H, and F genes of PPRV. The primer pairs M1/M2, H1/H2, and F1/F2 were used to amplify the M, H, and F genes, respectively. The underlined nucleotidesin the forward primers are a Kozak sequence added to optimize the expression of the target foreign gene. Restriction enzyme sites were introduced at the respective 5′-termini (shown in bold); the relevant enzymes are indicated in the last column. The primer pairs M13 For/M13 Rev were used to identify the recombinant bacmids.

The transfer plasmids were generated using the pFastBac Dual vector (Invitrogen, Grand Island, NY, USA), which contains two promoters. The M gene was cloned, as a1-kb*Xho*I*–Kpn*IDNA fragment, downstream of the p10 promoter within the pFastBacDual bacmid transfer vector that had been digested with *Xho*I and *Kpn*I. The resulting baculovirus transfer plasmid containing the PPRV M gene was designated p-M.

A bacmid transfer vector expressing both the M and H genes was prepared by cloning the H gene, as a 1.8-kb *Bam*HI*–Hin*dIII DNA fragment, into the *Bam*HI/*Hin*dIII sites downstream of the *Autographacalifornica* multiple nuclear polyhedrosis virus (AcMNPV) polyhedron(P_H_) promoter in p-M. The resulting plasmid, p-MH, encoded the M and H genes downstream from the p10 and P_H_ promoters, respectively.

**Figure 1:**
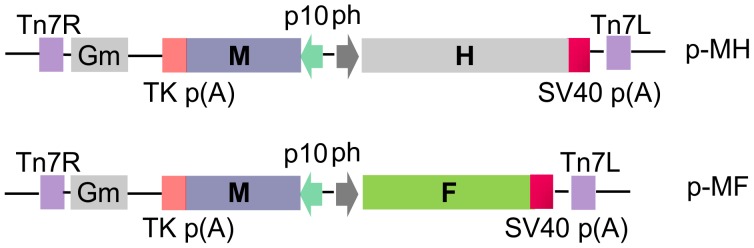
Schematic diagram and identification of recombinant pFastBac vectors. The M gene of peste des petits ruminants virus (PPRV) was inserted under the control of the baculovirus p10 promoter and the PPRVH or F genes were controlled by the baculovirusP_H_promoter, as described in the Materials and Methods section.

Similarly, a bacmid transfer vector,p-MF, which permitted expression of both the M and F genes, was prepared by cloning the F gene, as a 1.6-kb *Bam*HI–*Xba*I DNA fragment, downstream of the P_H_ promoter, into p-M (Fig.1).

### Generation of recombinant baculoviruses (rBVs)

Recombinant bacmids were produced by site-specific transpositioning after transformation of bacmid transfer plasmids containing the PPRV genes into *E.coli* DH10Bac competent cells(Invitrogen, Carlsbad, CA, USA), which contained the AcMNPV baculovirus genome. The recombinant bacmids were identified by PCR using M13 primers together with gene-specific primers (Table 1). The recombinant bacmid DNA was transfected into Sf21 insect cells using Cell Fectin II (Invitrogen, Grand Island, NY, USA), according to the Bac-to-Bac Expression Systems manual (Invitrogen, Grand Island, NY, USA). Briefly, a transfection mixture that contained 1 µg of recombinant bacmid DNA, 8µL of Cell fectinII, and 2 mL of unsupplemented Grace's Medium (Invitrogen, Grand Island, NY, USA), was prepared. Following incubation at room temperature for 30 min, the mixture was added to Sf21 insect cells, which had been seeded at 1×10^6^ cells per well, in 6-well plates. The transfection mixture and cells were incubated for 5 h at 28°C; the transfection mixture was then removed and 2 mL serum-free Sf-900II medium was added. The cultures were incubated at 28°C for 5 days and the supernatants were harvested as the first passage (P1) viral stock. Cells were continuously passaged in order to amplify baculovirus stock with the highest viral titer. The recombinant baculovirus stock was titrated by agarose plaque assay on Sf21 insect cell cultures in 6-well plates.

### Protein expression and sucrose gradient ultracentrifugation

To produce PPRV-H VLPs, Sf21 insect cells were infected in a 200-mL volume, at a cell density of 2×10^6^ cells per mL, with rBVs expressing PPRV H and M proteins, at a multiplicity of infection (MOI)of 4. Similarly, PPRV-F VLPs were produced by infecting Sf21 insect cells with rBVs expressing the PPRVF and M proteins. Cell culture supernatants were harvested 3 days post-infection, cleared by low-speed centrifugation (2000×*g*,30 min at 4°C)to remove cells, and VLPs in the supernatants were then pelleted by ultracentrifugation using a SorvallT1250 rotor (100,000×*g*, for 1 h, at 4°C). The resulting pellets were dissolved in 2 mL of phosphate-buffered saline solution (PBS, pH7.2), and were then further purified through a discontinuous sucrose gradient (20–30–60% w/v, in PBS) and sedimented by ultracentrifugation in a SorvallTH641 swinging bucket rotor (100,000×*g*, for 3 h, at 4°C). As a control, we used the PPRV Nig75/1 virus. The visible opalescent bands in the sucrose gradient were collected, diluted in PBS, and pelleted by centrifugation in a Sorvall T1250 rotor (100,000 ×*g*, for 2 h, at 4°C) to remove the sucrose. The sedimented particles were resuspended in PBS and stored at 4°C. The concentration of the VLPs was determined using a bicinchoninic acid protein assay kit (BCA protein assay kit; Beyotime, Shanghai, China).

### Detection of protein expression by western blot analyses

To detect the protein reactivity of VLPs, purified VLPs were boiled for 5 min in reducing Laemmli sample buffer and resolved by 10% sodium dodecyl sulfate-polyacrylamide gel electrophoresis, and then transferred to a polyvinylidene difluoride membrane by electroblotting (Bio-Rad, Hercules, CA, USA). The membrane was blocked with 5% dried non-fat milk in PBST (0.05% Tween-20 in PBS), overnight at 4°C, and then incubated with goat serum against PPRV (diluted 1∶200 in blocking buffer), for 1 h at 37°C. The membrane was washed three times with PBST, for 5 min each time, and then incubated with horseradish peroxidase (HRP)-conjugated rabbit anti-goat IgG antibody (diluted 1∶2000 in blocking buffer; Zhongshanjinqiao, Beijing, China). Following several washes with PBST, the specific bands were detected using the substrate 3,3′-diaminobenzidine tetrahydrochloride (Tiangen, Beijing, China) for 5 min at room temperature. The reaction was stopped with water and membranes were photographed.

### Transmission electron microscopy (TEM)

To examine budding of VLPs, Sf21 insect cells that had been infected with rBVs expressing the M and H/F proteins were fixed with 0.25% glutaraldehyde and 1% osmium tetraoxide, dehydrated with ethanol, and then embedded in SPI-PON 812 resin (Structure Probe, West Chester, PA, USA). Thin sections were stained with lead citrate and uranyl acetate and then observed by electron microscopy [21].

The presence and morphology of the purified VLPs and PPRV were evaluated by TEM of negatively stained preparations. A carbon-coated grid was set on a 20-µL sample drop for 10 min, and washed with deionized water. Washed samples were negatively stained with 1% uranyl acetate at pH 4.5. Excess stain was removed using filter paper, and the grids were observed at magnifications ranging from 60,000× to 200,000× on a transmission electron microscope (H-7500, Hitachi, Tokyo, Japan) operating at 80 kV.

### Immunization and sample collection

The designs of the animal experiments are shown in Table 2. The animal experiments were conducted with prior approval from the Animal Care and Use Committee of Chinese Academy of Agricultural Sciences, China. All animal experiments were carried out in accordance with the requirements of the Regulations of Experimental Animal Administration of the PR China. All efforts were made to minimize suffering. The proteins that were used to vaccinate animals were purified by sucrose gradient ultracentrigugation and the concentration was determined by using a BCA protein assay kit (BCA protein assay kit; Beyotime, Shanghai, China).

**Table 2:** Experimental designs used for the animal studies.

Experimental animals	Group	Number of animals	Vaccine	Route	Dosage (µg)	Dose	Time-point for sera collection
Mice	1	10	PPRV-H VLP	S.C.	10	2 doses, 4 weeks apart	1 week before immunization, 3 weeks after primary and booster immunization.
	2	10	PPRV-H VLP	S.C.	40	2 doses, 4 weeks apart	
	3	10	PPRV-F VLP	S.C.	10	2 doses, 4 weeks apart	
	4	10	PPRV-F VLP	S.C.	40	2 doses, 4 weeks apart	
	5	10	PPRV	S.C.	10	2 doses, 4 weeks apart	
	6	10	PPRV	S.C.	40	2 doses, 4 weeks apart	
	7	10	PBS	S.C.	/	2 doses, 4 weeks apart	
Goats	1	5	PPRV-H VLP	S.C.	300	2 doses, 3 weeks apart	2 week before immunization, 3 weeks post-primary inoculation and 2,4,8, or 12 weeks post-booster inoculation.
	2	5	PPRV-F VLP	S.C.	300	2 doses, 3 weeks apart	
	3	5	PPRV	S.C.	10^5.5^TCID_50_	2 doses, 3 weeks apart	
	4	5	PBS	S.C.	/	2 doses, 3 weeks apart	

PBS  =  phosphate-buffered saline; PPRV  =  Peste des petits ruminants virus; S.C.  =  subcutaneous, TCID_50_  = 50% tissue culture infective dose; VLP  =  virus-like particles.

For mouse experiments, female inbred BALB/c mice (Vital River, Beijing, China), aged 6 to 8 weeks, were randomly divided into seven groups (n = 10 for each group). Mice were immunized subcutaneously (s.c.) with different amounts (10 or 40 µg total protein per mouse) of PPRV VLPs or PPRV Nig75/1diluted in 100µL of PBS. All groups received a booster injection of the original dosage at 4 weeks after the primary immunization. No adjuvant was used for any of the mice. Blood samples were collected by retro-orbital plexus puncture, at various time-points. After the blood samples were allowed to clot and then centrifuged, serum samples were collected and stored at −20°Cuntil required for antibody titration.

For goat experiments, 20 outbred goats (6–24 months of age) were randomly divided into four groups of five each, and housed separately. None of the goats had detectable levels (titers less than1∶5) of PPRV neutralizing antibodies. Groups 1 and 2 were vaccinated subcutaneously with 300 µg PPRV-H or PPRV-F VLPs diluted in 1 mL of PBS, respectively. Group 3 goats were inoculated with 1 mL PPRV Nig75/1(10^5.5^ TCID_50_), as positive control. Group 4 goats were inoculated subcutaneously with 1 mL PBS as negative control. Booster vaccination was performed using the same dose, at 3 weeks after primary immunization. Serum samples were collected at various time-points post-immunization, and stored at −20°C until analyzed.

### ELISA for PPRV-specific antibody

Sera from immunized and control mice were analyzed for PPRV-specific antibodies (IgG, IgG1, and IgG2a) by indirect ELISA. Briefly, 96-well flat-bottomed plates were coated with 100µLof purified PPRV whole-virus antigen at a concentration of 2 µg/mL per well in 0.1 M carbonate/bicarbonate coating buffer (pH 9.6),overnight at 4°C, and then the plates were blocked with 5% skim milk in PBST, for 2 h at 37°C. Serum samples, serially diluted in PBST, were added to the wells, in duplicate, and incubated at 37°C for 1 h. After washing with PBST three times, each well received 100µL of a 1/4000-diluted HRP-labeled goat anti-mouse IgG, IgG1, and IgG2a (Southern Biotech, Birmingham, AL, USA) and incubated at 37°C for 1 h. Finally, after washing with PBST three times, 100 µL of freshly prepared tetramethylbenzidine (Tiangen, Beijing, China) solution was added to each well and color was allowed to develop for 10 min, before stopping the reaction with 100µL of 2 M H_2_SO_4._Optical density (OD) values were measured at a wavelength of 450 nm using an ELISA microplate reader (Multiskan Ascent; Thermo, Vantaa, Finland). The mean titers were calculated from log_10_ of the highest serum dilution that yielded a two-fold higher OD value compared to that of sera from PBS-vaccinated mice.

### Virus neutralization test (VNT)

All serum samples from immunized mice and goats were analyzed for the detection of PPRV-specific virus neutralization antibody (VNA) titers using a micro-neutralization assay, according to the 2013 OIE terrestrial manual[22]. Briefly, serum samples were complement-inactivated at 56°C for 30 min and were then serially diluted two-fold (mice sera, from 1∶4; goat sera, from 1∶5) in cell culture medium. The diluted sera (100µL) were mixed with an equal volume of PPRV (1000TCID_50_/mL) in a 96-well cell culture plate and incubated at 37°C for 1 h; then, 50µL of Vero cell suspension was added to each well and the cells were cultured at 37°C in the presence of 5% CO_2_.A series of control wells for virus and uninfected cells were also included. Cells were observed for a cytopathic effect (CPE) by microscope at day 8 after incubation, and the VNA titer was calculated. VNA titer was defined as the highest dilution of the test serum at which the CPE was inhibited by at least 50%.

### Lymphocyte proliferation assay

Three mice from different groups were sacrificed at 7 days after booster immunization and splenocyte suspensions were prepared as previously described[23]. Splenocytes were seeded in 96-well flat-bottom plates at a density of 2×10^6^cells per well in RPMI1640 medium (Invitrogen, Grand Island, NY, USA) containing 10% FBS. Splenocytes were stimulated in vitro with 50µL of UV-inactivated PPRV Nig75/1(10^4^ TCID_50_). Concanavalin A (Sigma-Aldrich, St. Louis, MO, USA) was used as positive control, and medium was used as negative control. After 3 days' incubation at 37°C in a 5% CO_2_atmosphere, the proliferative response was determined using a 3-(4,5-dimethylthiazol-2-yl)-5-(3-carboxymethoxyphenyl)-2-(4-sulfophenyl)-2H-tetrazolium, inner salt (MTS) assay, using the Cell Titer 96 Aqueous One Solution Cell Proliferation kit (Promega, Madison, WI, USA), following the manufacturer's instructions. The lymphocyte proliferation rate was quantified using the stimulation index (SI), which was calculated as the ratio of the OD_490_ of the stimulated cells to the OD_490_ of the negative controls.

Goats were bled via jugular vein puncture at week 2 after the booster injection. Peripheral blood mononuclear cells were prepared as previously described [10]. Cells(2×10^5^ cells per well) were plated in 96-well plates and stimulated with inactivated PPRV Nig75/1 for 72 h at 37°C under 5% CO_2_. Unstimulated cells, cultured in medium alone, were used as negative control. The proliferative response was determined by MTS assay, as described above.

### Interferon (IFN)-γELISPOT assays

Splenocytes from inoculated mice were obtained and plated at a density of 2×10^5^ cells per well in a 96-well ELISpot plate, which had been coated with IFN-γ**.** One hundred microliters of inactivated PPRV Nig75/1was added as stimulus. The cultures were then incubated in a 37°C humidified incubator, with 5% CO_2_, for 16 h, and then spots were detected as per the manufacturer's instructions (Mabtech, Nacka, Sweden).

### Statistical analysis

The data were analyzed with one-way ANOVA in GraphPad Prism version 5.00 (GraphPad Software, San Diego, CA, USA); p-values less than 0.05 were considered statistically significant.

## Results

### Generation and identification of recombinant baculovirus

The open reading frames of PPRV M, H, and F genes from PPRV strain Nig75/1 were amplified by RT-PCR and sub-cloned into pFastBacDual vectors (Fig.1).The insertion of the genes into the expression vector was confirmed by enzyme digestion analysis and DNA sequencing. The rBVs were generated after transfection of Sf21 insect cells with recombinant bacmids. The titers of rBVs-PPRV MH and rBVs-PPRV MF were titrated in a plaque-forming assay, and exhibited titers of 3.5×10^7^ and 4.7×10^7^PFU/ml, respectively, at the third passage.

### Production and characterization of VLPs

**Figure 2:**
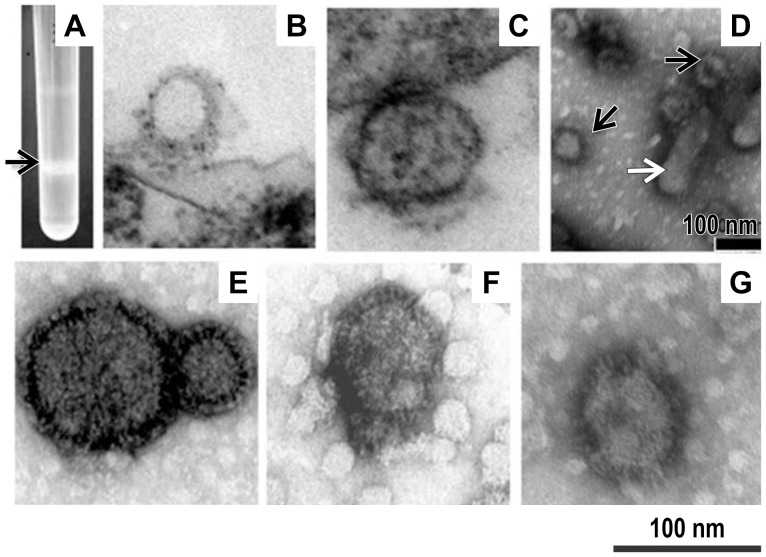
Photograph of a sucrose gradient and transmission electron microscopy (TEM) of virus-like particles (VLPs). The culture supernatant of Sf21 insect cells at 72-h post-infection with recombinant baculoviruses (rBVs) was subjected to sucrose gradient centrifugation; the opalescent band (A, indicated by the arrow) was collected for TEM after staining with 1% uranyl acetate. a trace of residual baculoviruses(D, indicated by the white arrow) were consistently detected in VLP preparations(D, indicated by the black arrow). Both PPRV-H VLPs (F) and PPRV-F VLPs (G) isolated in this manner showed spherical shapes upon TEM, with spikes on their surfaces, and with a diameter of approximately 80–100 nm were observed. Native peste des petits ruminants virus (PPRV) propagated in Vero cells was also purified and negatively stained (E). Ultrathin sections of Sf21 insect cells showing budding of PPRV-H and PPRV-F VLPs (B and C) from the plasma membrane at 48 h after infection with rBVs.

PPRV-H VLPs and PPRV-F VLPs were produced and purified as described in the Materials and Methods section. The VLPs bands were mainly distributed between 30% and 60%sucrose in the gradient after ultracentrifugation (Fig.2A). Electron microscopic examination of negatively stained samples revealed that both PPRV-H and PPRV-F VLPs showed spherical shapes, with spikes on their surfaces, and with a diameter of approximately 80–100 nm, which is similar to the morphology and size of native PPRV particles propagated in Vero cells(Fig.2E–G).An ultrathin section of Sf21 insect cells infected with rBVs at 48-h post-infection was investigated, revealing VLPs with a diameter of approximately 100 nm budding from the plasma membrane, and some spikeless VLPs distributed throughout the cytoplasm(Fig. 2B–C).

**Figure 3:**
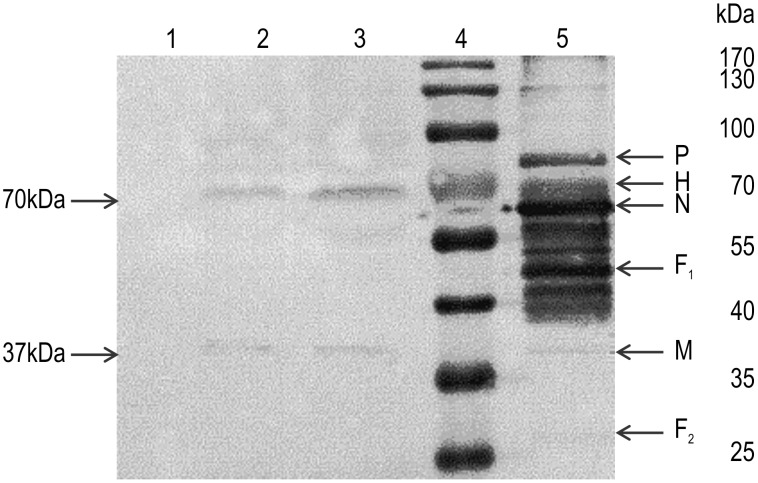
Expression of the M, H, and F proteins from peste des petitsruminants virus (PPRV) and virus-like particles (VLPs). TheVLPs were purified using sucrose gradient centrifugation, and the protein composition was determined by western blot analysis using a mouse anti-PPRV polyclonal antibody. Lane 1, Sf21 cells (negative control);lane 2, PPRV-H VLPs; lane 3,PPRV-F VLPs; lane 4, molecular weight marker;lane 5, PPRV (positive control).

Incorporation of the H or F proteins, as well as M protein, into VLPs was confirmed by western blotting using anti-PPRV antibodies, by the presence of 37 kDa, 70 kDa, and 70 kDa bands, corresponding to the M, H, and F proteins, respectively, on the blots (Fig.3). The sizes of the M and H proteins corresponded to those predicted from the deduced protein sequences, but the F protein band derived from VLPs differed from the form of PPRV particles propagated in mammalian cells. In the mammalian cell expression system, the F protein is synthesized as a precursor (F0) of about 60 kDa; after translation, F0 is glycosylated and cleaved to yield a 46-kDa (F1) and 25-kDa (F2) disulfide-link-competent F protein. In the baculovirus–insect cell expression systems, the PPRV F protein was generated as a 70-kDa band in the western blot membrane, indicating that the F protein was not cleaved and was generated as the F0 precursor, which was also glycosylated. In total, the amount of the expressed VLPs was about 3-4.5 mg/L in the cell culture medium.

### PPRV-specific antibody responses in immune serum

**Figure 4:**
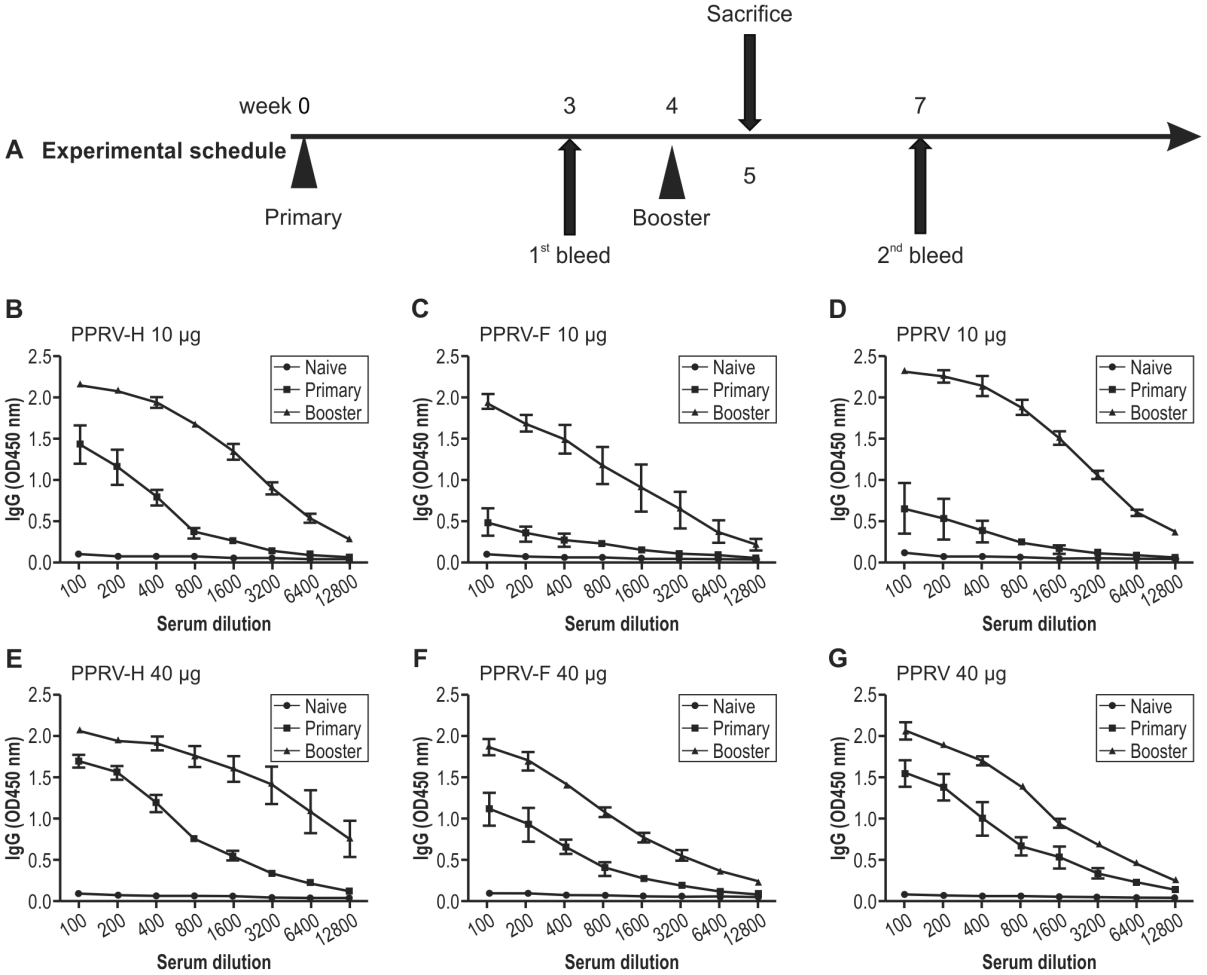
Experimental schedule and peste des petitsruminants virus (PPRV)-specific IgG antibody responses to PPRV-H or PPRV-F virus-like particles (VLPs). Groups of mice were immunized subcutaneously, twice, as indicated, with 4-week intervals. Three mice from each group were sacrificed and splenocytes were pooled for the lymphocyte proliferation assay. Enzyme-linked immunosorbent assay (ELISA) plates were coated with whole PPRV, as indicated in the Materials and Methods section. Serially diluted sera were used after primary and booster vaccinations. Total immunoglobulin G levels were determined from mice immunized with PPRV VLPs or PPRVNig75/1.

The specific antibody responses of mice immunized with PPRV-H VLPs or PPRV-F VLPs were determined by indirect ELISA. The levels of total PPRV-specific IgG in the serum after primary and booster vaccinations were determined; the results are shown in Fig.4. Total IgG titers in all groups of mice immunized with VLPs or PPRV were higher after the booster than after the primary vaccination, indicating that booster immunization could stimulate the immune memory cells and induce an anamnestic response, promoting antibody production and exaltation. When mice were immunized with a lower antigen dose of 10 µg, total IgG titers of mice immunized with PPRV-H VLPs were significantly higher than those of mice immunized with PPRV-F VLPs or PPRV after the primary vaccination, indicating that the titers of antibodies against PPRV-H VLP proteins increased faster than the titers of antibodies against PPRV-F VLPs or PPRV. The maximal titers detected in both VLP- and virus-immunized mice were similar after the booster vaccination. Similar patterns of total IgG responses were found in mice immunized with 40 µg VLPs or PPRV.

**Figure 5:**
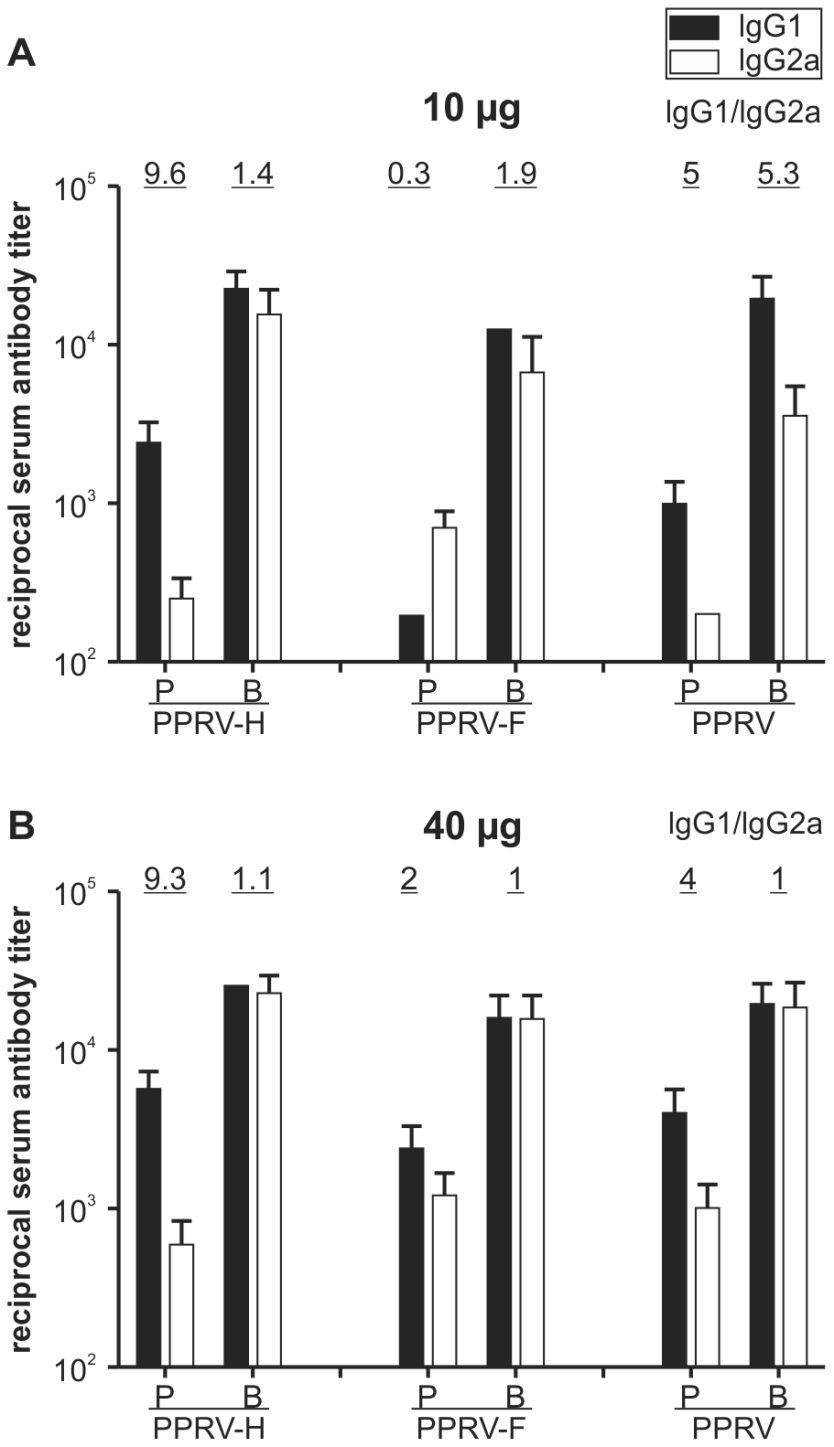
Peste des petitsruminants virus (PPRV)-specific serum IgG1 and IgG2a antibody responses. Sera were taken from immunized mice at 3 weeks after primary (P) and booster (B) inoculation. PPRV-specific IgG1(black columns) or IgG2a(white columns) antibodies were determined by enzyme-linked immunosorbent assay (ELISA). The ratios of IgG1 to IgG2a subclasses are given above the columns. Bars indicate standard deviations.

PPRV-specific serum antibodies of IgG1 and IgG2a were also evaluated by ELISA. These results demonstrated that mice immunized with PPRV-H VLPs and PPRV using a lower dose of antigen (10 µg) induced high IgG1 serum antibody titers(1∶1000 to 1∶2400), and lower specific IgG2a serum antibody titers(1∶200 to 1∶250) after the first immunization. In contrast, the levels of IgG1 antibody responses were lower(1∶200),and the IgG2a responses were higher (1∶700),in mice immunized with PPRV-F VLPs relative to those observed in mice immunized with PPRV-H VLPs or PPRV. The levels of IgG1 and IgG2a from all three immunized groups of mice increased after the booster immunization (Fig.5A). Similar patterns of IgG1 and IgG2a responses, as well as IgG1:IgG2a ratios, were found in mice immunized with 40 µg VLPs or PPRV (Fig.5B).

Taken together, the data indicated that immunization with PPRV-H VLPs induced predominantly specific IgG1 serum antibodies, favoring the stimulation of a T-helper class 2(Th-2) immune response against PPRV, while immunization with PPRV-F VLPs induced a stronger Th-1 immune response, despite the use of a lower immunization dose.

### Neutralizing antibody responses

Since the neutralizing activity against PPRV is an indicator of functional antibodies that confer protective immunity, we investigated whether mice and goats immunized with PPRV VLPs could produce neutralizing antibodies, using a micro-neutralization assay. Serially diluted sera from various time-points post-immunization were complement-inactivated, and incubated with live PPRV Nig75/1; the CPE assays were conducted in Vero cells. As shown in Table3, PPRV-H or PPRV-F VLPs-vaccinated mice developed VNA, and the titers ranged from 1∶32 to 1∶64 and from 1∶16 to 1∶32, after booster immunization. As shown in Table 4, at 2 weeks after primary immunization, the titers of VNA in the PPRV-H VLPs- and PPRV-vaccinated goat groups exceeded 10, a number that represents a margin considered to be protective in goats vaccinated with live attenuated PPRV vaccines [22]. Moreover, this value was significantly enhanced after the booster immunization and reached values of 80–160; these high VNA titers were maintained for at least 6 weeks, and decreased gradually from 15 weeks after primary immunization. The titer of the PPRV-F VLPs group was lower compared to that of the PPRV-H VLPs- and PPRV-vaccinated goat groups. However, 2weeks after the booster immunization, all the goats that had been immunized with PPRV-F VLPs showed VNA titers above 10.

**Table 3:** Peste des petitsruminants virus (PPRV) neutralizing antibody titers and lymphocyte proliferation responses in mice immunized with PPRV virus-like particles (VLPs) or native PPRV particles.

Immunogen (µg)	Neutralization titer (weeks post-primary immunization)			Mean Stimulation Index*
	0	3	7	
PPRV-H VLP(10)	<4	8–16	32–64	1.98±0.06^a^
PPRV-H VLP(40)	<4	16–32	32–64	1.73±0.12^ a^
PPRV-F VLP(10)	<4	4–8	8–16	1.81±0.34^ a^
PPRV-F VLP(40)	<4	8–16	16–32	1.87±0.20^ a^
PPRV (10)	<4	4–16	32–64	1.87±0.23^ a^
PPRV (40)	<4	16–32	32–64	1.78±0.14^ a^
PBS	<4	<4	<4	1.07±0.15 ^b^

PPRV neutralizing antibody titers was determined in mice immunized with PPRV VLPs and PPRV vaccine virus Nig75/1, and the serum samples were collected at weeks 0, 3, and 7 following the primary immunization. Lymphocyte proliferative responses were analyzed using splenocytes collected at 1 week after boosting.

PBS  =  phosphate-buffered saline, as negative control; PPRV  =  Peste des petits virus; VLP  =  virus-like particles.

*Different superscript letters indicate that the differences were significant (p<0.05), same superscript letters indicate that the differences were not significant (p>0.05). (according to one-way ANOVA, using GraphPad).

**Table 4:** Peste des petitsruminants virus (PPRV) neutralizing antibody titers and lymphocyte proliferation responses in goats immunized with PPRV virus-like particles (VLPs) or native PPRV particles.

Group	Neutralization titer (Weeks post primary immunization)								Mean stimulation Index*
	0	1	2	3	5	7	11	15	
PPRV-H VLP	<5	5–10	20–40	40–80	80–160	80–160	160	80–160	2.44±0.20^a^
PPRV-F VLP	<5	<5	5–10	5–20	10–80	40–160	40–80	40–80	2.32±0.18^a^
PPRV	<5	5–10	20–80	40–80	80–160	160–320	160–320	160–320	2.61±0.32^a^
PBS	<5	<5	<5	<5	<5	<5	<5	<5	1.21±0.10^b^

PPRV neutralizing antibody titers in goats immunized with the PPRV VLPs, PPRV, and PBS. Serum samples were collected at weeks 0,1,2, 3, 5, 7, 11, and 15 after primary vaccination. Proliferation responses of peripheral blood lymphocytes in goats were tested at week 2 after boost immunization.

PBS  =  phosphate-buffered saline, as negative control; PPRV  =  Peste des petits virus; VLP  =  virus-like particles.

*Different superscript letters indicate that the differences were significant (p<0.05), same superscript letters indicate that the differences were not significant (p>0.05). (according to one-way ANOVA, using GraphPad).

Taken together, the data of the mouse and goat vaccination experiments showed that serum VNA levels were significantly higher in the PPRV-H VLPs-immunized group than in the PPRV-F VLPs-immunized group, and were comparable to that of the whole virus-immunized group. No neutralization activity against PPRV was detected when using pre-immune serum or in the sera of experimental animals injected with PBS.

### Cellular immune responses

To investigate the cell-mediated immune responses induced by VLPs, the lymphocyte proliferation response of mice was analyzed after the booster immunization. As shown in Table3, compared with the PBS control group, mice immunized with VLPs or PPRV exhibited significantly stronger lymphocyte proliferation responses (P<0.05). As shown in Table4, the data from the lymphocyte proliferation assay of goats from the three groups immunized with PPRV-H VLPs, PPRV-F VLPs, or attenuated PPRV indicated similarly strong cellular immune responses. There was no significant difference among mice immunized with different concentrations of VLPs or PPRV (P>0.05).

**Figure 6:**
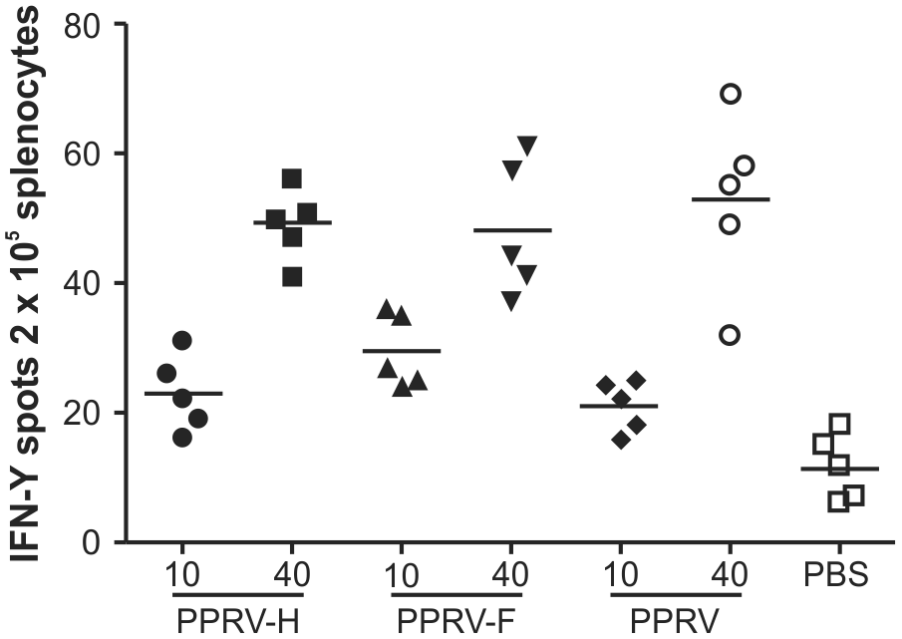
Specific interferon (IFN)-γproduction in response to peste des petits ruminants virus (PPRV) proteins in splenocytes from mice, as detected by ELISPOT assay. Splenocytes from mice inoculated with PPRV virus-like particles (VLPs), PPRV per se, or phosphate-buffered saline (PBS) were isolated and cultured for 16h in the presence of inactivated PPRV. The production of IFN-γwas measured using an ELISPOT assay. Samples were tested in triplicate and mean IFN-γvalues for each mouse are shown.

We also evaluated the mounting of PPRV-specific T-cell responses in vaccinated mice. Splenocytes, obtained at 7days after the booster immunization, were cultured in the presence of inactivated PPRV, and IFN-γ production was measured by ELISPOT assays (Fig.6). Both PPRV-H VLPs- and PPRV-F VLPs-vaccination induced specific IFN-γ production in response to PPRV exposure; the frequency of cells secreting IFN-γ positively correlated with the immunization dose. No specific production of IFN-γ in response to PPRV exposure was detected in control mice inoculated with PBS.

Altogether, these data demonstrated that inoculation with PPRV-H VLPs and PPRV-F VLPs can generate specific T-cells that recognize the PPRV H and F proteins in vivo.

## Discussion

The minimum molecular requirement for efficient assembly and release of PPRV virions is still unknown. In this study, we found that the major structural genes of PPRV can be efficiently expressed in recombinant baculovirus-infected Sf21 insect cells and that these genes can then support the production of PPRV VLPs. The expression of M protein was necessary and sufficient for the formation of VLPs (data not shown); the additional expression of the H or F glycoproteins allowed formation of budding particles with the typical morphology and size of PPRV and related paramyxoviruses. A recent study demonstrated that PPRV spikeless VLPs could be formed by co-expression of the M and N proteins in insect cells; however, expression of M protein alone in insect cells did not result in VLP formation, as assessed by TEM [24], which was contrary to our results. The finding that M protein alone could be released from cells and generate VLPs has also been observed for some other paramyxoviruses, including human parainfluenza virus type1 [25], Sendai virus [26], Newcastle disease virus [27], and Nipha virus [28], as well as for viruses in other related virus families, including Ebola [29] and vesicular stomatitis virus [30].In addition, some paramyxovirus M proteins, such as parainfluenza virus type5 [31]and mumps virus [32], lack the ability to direct efficient VLP production when expressed alone in cells, but VLP production becomes highly efficient when M proteins are expressed together with other viral proteins. Hence, although the requirements for efficient VLP production differ among paramyxoviruses, it appears as if, in general, M protein is the major requirement for paramyxovirus VLP production, as VLPs cannot be formed in its absence [6], [33].

The baculovirus expression vector–insect cell system has been utilized extensively for VLP production, and particularly for production of enveloped VLP vaccines [34], [35], [36]. Baculovirus expression of heterologous genes permits multiple post-translational modifications, like folding, oligomerization, phosphorylation, glycosylation, acylation, disulfide bond formation, proteolytic cleavage, and so on, which are similar or identical to those that occur in mammalian cells. However, in this study, the molecular size of the expressed F protein was 70 kDa, but the F protein was cleaved into F1 and F2 protein upon expression in mammalian cells; the results indicated that there are differences in the post-translational modification in the two expression systems. Furthermore, insect cells are able to grow in serum-free medium cultures; this is highly desirable, because serum may be a cause of adventitious viruses. Therefore, its use requires more intensive purification processes to remove serum proteins. Additionally, these cells are perfectly adapted to suspension conditions, allowing for large-scale culture [37], [38]. These advantages make the baculovirus expression vector–insect cell system a promising platform for production of VLPs[39].

In this study, we chose the pFastBacDual (Invitrogen) baculovirus transfer vector, which contains two promoters, so that insect cells infected with a single recombinant bicistronic baculovirus could express two proteins concurrently. The co-expression strategy is particularly important for the efficient assembly of multi-protein complexes, where simultaneous expression of multiple viral proteins is essential[40].Co-expression and co-infection strategies have previously been compared in the production of triple-layered rotavirus VLPs(VP2,VP6, and VP7), and SARS VLPs. For production of rotavirus VLPs, co-expression was more efficient than co-infection[41]. SARS VLPs could not be generated in Sf9 cells co-infected with three recombinant viruses expressing the M, E, and S proteins, but spontaneous assembly of highly stable VLPs occurred when using a single recombinant baculovirus expressing all three SARS proteins simultaneously[35].

The main limitation of the baculovirus expression vector–insect cell system is the co-production of recombinant baculoviruses (enveloped viruses) during the infection process; thus, when developing PPRV VLP vaccines, the challenging task is the purification of VLPs from the recombinant baculoviruses, which share a similar density and enveloped structure [39], [42]. In our study, although purification was possible by centrifugation in sucrose density gradients, residual baculoviruses were consistently detected in VLP preparations (Fig. 2D).Given the safety concerns, VLPs should be purified via chemical (binary ethylenimine) or physical(UV irradiation) inactivation treatments, to eliminate any possible baculovirus infectivity when considering clinical trials.

For many viruses, recombinant VLPs have been shown to yield promising vaccines, because of their robust immunogenicity, elicitation of protective neutralizing antibodies (due to the presence of conformational epitopes on VLPs), and their safety profiles (as they do not contain any viral genetic material). In this study, the effectiveness of PPRV VLPs as an immunogen was demonstrated in mice and goats. The results indicated that high specific antibody titers against PPRV could be induced by both PPRV-H VLPs and PPRV-F VLPs, which were as high as those resulting from immunization with PPRVNig75/1. The VNA titers elicited by PPRV-H VLPs were much higher than those induced by PPRV-F VLPs, and the VNA titers of PPRV-H VLPs were as high as the responses to the attenuated virus vaccine. Previous reports have shown that the H protein of PPRV is a more potent inducer of high VNA titers than the F protein[11], while the F protein seems to induce mainly a cellular immune response[43].Our results demonstrated that mice immunized with PPRV-H and PPRV-F VLPs exhibited a similarly high level of cell-mediated immune responses.

We further showed that subcutaneous immunization of mice with PPRV-H VLPs at a dose of 10 µg, in the absence of adjuvant, induced strong humoral immune responses. This immunogenicity is related to the structural features of the VLPs, which are particulate structures that contain a dense and repetitive pattern of epitopes on their surfaces. In contrast, monovalent, non-particulate antigens do not always reflect the native structure of the protein, and the related antibody responses are not always specific for physiologically relevant epitopes. Consequently, administration of these vaccines often requires use of strong adjuvants, as well as large and frequent doses of the antigen. In contrast, VLP-based vaccines are strongly immunogenic, often reducing or eliminating the need for exogenous adjuvants[44], [45]. An additional benefit of this VLP-vaccination approach is that it may allow improved surveillance through DIVA, as seroconversion to N protein will occur only in animals that have been infected with PPRV, but not in VLP-vaccinated animals that have not been infected. A commercially available N protein-based competitive ELISA kit might strengthen the usefulness of VLP vaccines[46]. We also detected PPRV-specific antibodies in the vaccinated goats, using an N protein-based competitive ELISA established in our laboratory. No N protein-specific antibodies were found in goats vaccinated with PPRV H or PPRV F VLPs; in contrast, seroconversion to N protein was detected in goats vaccinated with the PPRV Nig 75/1 attenuated virus (data not shown).

To our knowledge, this is the first report that demonstrated the construction of PPRV VLPs consisting of immunogenic proteins and that assessed their immunogenicity in two animal models, viz., mice and goats. Taken together, the results of our study suggested that PPRV VLPs can be generated in insect cells simultaneously expressing the PPRV M protein with H or F protein. The VLPs had a morphology and size similar to those of native virus particles. Humoral and cellular immunity against PPRV were efficiently induced in mice and goats vaccinated with these PPRV VLPs, without the need for adjuvants. Thus, PPRV-H VLPs or PPRV-F VLPs may be promising vaccine candidates for PPR, and DIVA tests may prove of great benefit in future control programs for this disease.
